# Empirical optimization of risk thresholds for dengue: an approach towards entomological management of *Aedes* mosquitoes based on larval indices in the Kandy District of Sri Lanka

**DOI:** 10.1186/s13071-018-2961-y

**Published:** 2018-06-28

**Authors:** Lahiru Udayanga, Nayana Gunathilaka, Mohamed Cassim Mohamed Iqbal, Mohamed Mujithaba Mohamed Najim, Kusumawathie Pahalagedara, Wimaladharma Abeyewickreme

**Affiliations:** 1 Molecular Medicine Unit, Faculty of Medicine, University of Kelaniya, Ragama, Sri Lanka; 2 Department of Biosystems Engineering, Faculty of Agriculture & Plantation Management, Wayamba University of Sri Lanka, Makadura, Sri Lanka; 3 Department of Parasitology, Faculty of Medicine, University of Kelaniya, Ragama, Sri Lanka; 4 National Institute of Fundamental Studies, Kandy, Sri Lanka; 5 Department of Zoology and Environment Management, Faculty of Medicine, University of Kelaniya, Kelaniya, Sri Lanka; 6 Anti Malaria Campaign, Regional Office, Kandy, Sri Lanka; 7 Department of Parasitology, Faculty of Medicine, Sir John Kotelawala Defense University, Rathmalana, Sri Lanka

**Keywords:** Dengue, Empirical, Larval indices, Risk thresholds, Sri Lanka

## Abstract

**Background:**

Larval indices such as Premise Index (PI), Breteau Index (BI) and Container Index (CI) are widely used to interpret the density of dengue vectors in surveillance programmes. These indices may be useful for forecasting disease outbreaks in an area. However, use of the values of these indices as alarm signals is rarely considered in control programmes. Therefore, the current study aims to propose threshold values for vector indices based on an empirical modeling approach for the Kandy District of Sri Lanka.

**Methods:**

Monthly vector indices, *viz* PI, BI and CI, for *Aedes aegypti* and *Aedes albopictus*, of four selected dengue high risk Medical Officer of Health (MOH) areas in the Kandy District from January 2010 to August 2017, were used in the study. Gumbel frequency analysis was used to calculate the exceedance probability of quantitative values for each individual larval index within the relevant MOH area, individually and to set up the threshold values for the entomological management of dengue vectors.

**Results:**

Among the study MOH areas, Akurana indicated a relatively high density of both *Ae. aegypti* and *Ae. albopictus*, while Gangawata Korale MOH area had the lowest. Based on *Ae. aegypti*, threshold values were defined for Kandy as low risk (BI_agp_ > 1.77), risk (BI_agp_ > 3.23), moderate risk (BI_agp_ > 4.47) and high risk (BI_agp_ > 6.23). In addition, PI > 6.75 was defined as low risk, while PI > 9.43 and PI>12.82 were defined as moderate and high risk, respectively as an average.

**Conclusions:**

Threshold values recommended for *Ae. aegypti* (primary vector for dengue) along with cut-off values for PI (for *Ae. aegypti* and *Ae. albopictus*), could be suggested as indicators for decision making in vector control efforts. This may also facilitate the rational use of financial allocations, technical and human resources for vector control approaches in Sri Lanka in a fruitful manner.

**Electronic supplementary material:**

The online version of this article (10.1186/s13071-018-2961-y) contains supplementary material, which is available to authorized users.

## Background

Dengue fever, which is primarily transmitted by *Aedes aegypti* and *Aedes albopictus*, signifies approximately 390 million infections globally per annum [1, 2]. The most severe outbreak of dengue recorded in Sri Lanka, since its initial epidemic incidence in 1989, was witnessed in 2017 with 186,101 suspected cases, resulting an incidence rate of 888.31 patients per 100,000 people [3]. Notable allocations of financial, professional (human), medical and infrastructural resources are being made for the management of dengue patients and vector control, due to the severity of current dengue outbreaks in Sri Lanka.

With the absence of well-established and licensed vaccines or specific therapeutic cures, controlling of dengue vector populations remains the only way to prevent dengue transmission [4, 5]. Therefore, vector controlling entities (VCE) in Sri Lanka are mainly focused on the source reduction of *Aedes* mosquitoes, by eliminating potential breeding and resting habitats from the domestic environment *via* source reduction and chemical fogging. For this, , routine entomological surveys are conducted by the VCE of Sri Lanka at sentinel sites, by employing trained entomological field staff. Furthermore, vector control programmes attempt to encourage the general public to maintain vector free environments through elimination of breeding sites, *via* periodic awareness, implementation of dengue weeks for environmental cleaning and strengthening existing legislations against vector breeding in Sri Lanka. During the peak of an epidemic, chemical fogging is conducted based on the Breteau Index (BI) in dengue high risk areas. The selection of high risk areas to initiate vector control activities and post-monitoring of the effectiveness of ongoing vector control activities are achieved based on the entomological indicators, specifically larval indices in Sri Lanka [6].

Dengue virus infected adult females of *Aedes* mosquitoes that have completed the extrinsic incubation period are responsible for disease transmission. Hence, routinely executed vector surveillance is an effective tool in controlling dengue outbreaks based on the density and spatial dynamics of *Aedes* vectors [4, 6]. A variety of indices that mainly focus on the immature stages of the dengue vector, often known as *Stegomyia* indices, have been heavily utilized by majority of the developing countries for their routine surveillance activities since 1920 [7–9]. However, many studies have questioned the reliability of the use of *Stegomyia* indices that only focus on the immature stages of the vector, rather than concentrating on the actual density of the adult vector populations, which directly contributes to the transmission of the virus [8, 10, 11]. Limitations in the sensitivity of *Stegomyia* indices, methodological deficiencies, underestimations arising due to overlooked breeding sites and poor correlation between the larval indices and the adult densities have been highlighted as the key issues relevant to the use of *Stegomyia* indices [8, 11]. On the contrary, longitudinal analysis of *Stegomyia* indices have indicated notable associations with the dengue infections [4, 12]. However, factors such as limitations in resources (both human and financial), constraints in time and easy applicability have influenced many developing counties, including Sri Lanka, to still rely upon the traditional larval indices, namely Premise Index (PI), Breteau Index (BI) and Container Index (CI), in routine entomological surveillance activities, despite the above limitations in reliability and sensitivity [4, 7, 11]. Therefore, reliable threshold values that are capable of reflecting the incidence of dengue epidemics are essential for these larval indices to facilitate the management of dengue outbreaks.

Unfortunately, such critical cutoff values have rarely been determined for the above larval indices to enable efficacious implementation of precautionary actions for upcoming dengue epidemics [4, 9, 11–13]. Defining a critical vector density, below which dengue would not occur, or reliable threshold values to be followed in guiding vector control activities is a difficult and complex issue [4, 11]. A limited number of studies have attempted the setting up of threshold values for the most commonly utilized larval indices in different parts of the world. A variety of approaches ranging from statistical tools such as receiver operating characteristic curves (ROC) to geo-informatics based approaches, have been utilized for deriving of cut-off values for dengue epidemic management [4, 14–16]. For instance, several studies conducted by Moore et al. [16] in Puerto Rico, Perez et al. [15] in Havana and Tran et al. [14] in French Guiana, have used temporal graphics to compare the vector densities of confirmed infections. Regardless of the approaches used, the thresholds developed for the larval indices for management of dengue epidemics are considered to be less effective and sometimes remain poor in predicting the incidence of dengue epidemics [8, 11, 17]. For instance, dengue epidemics have occurred in Singapore, even when the national overall House Index (HI) was maintained < 1%, while another study in Brazil has indicated that no outbreaks of dengue occurred when the HI was < 1% [18, 19]. The threshold values of vector indices are influenced by a variety of factors such as geographical features, nature of the vector population, environmental factors (vegetation, meteorological factors, land-use practices, etc.) and characteristics of the human population (herd immunity, human migration status and cultural practices) within the region, making it practically difficult to establish critical thresholds for larval indices for dengue epidemic management [4, 20–23].

In the Sri Lankan context, the BI is mainly considered as the decision making parameter for vector control programmes. The BI value of 5 remains as the lowest threshold, while a scenario where the BI value > 5 with reported dengue cases or BI > 20 even without any dengue case, is recommended to be dealt with by chemical fogging [24]. Yet, this national guideline, which may have been adopted from thresholds developed for yellow fever in 1920 [11], often fails in addressing dengue epidemics due to local dynamics in the vector populations. Therefore, the current study was conducted with two major objectives: (i) to determine the natural distribution of vector densities (in terms of larval indices) at the regional level; and (ii) to establish threshold values to reflect the incidence of dengue outbreaks to guide epidemic management *via* effective entomological management of dengue vectors, based on an empirical modelling approach for the district of Kandy, Sri Lanka. The defined thresholds are expected to assist responsible VCEs of Sri Lanka to guide and coordinate community based vector control activities for management of dengue vectors and thereby control epidemic outbreaks and implement necessary precautions in order to minimize the risk of potential dengue outbreaks.

## Methods

### Study area

The Kandy District (69°33'36" to 70°17'24"N, 80°0'0" to 80°15'0"E), was selected as the study area. The district is divided into 20 regional local government institutions, including 23 regional health administrative divisions known as Medical Officer of Health (MOH) areas. A multi-cultural population of 1,369,899 resides within Kandy District that extends over an area of 1940 km^2^, resulting a population density of 710/km^2^ [25]. Kandy remains as one of the major tourist attractions due to its natural location, historical and religiously important places, increasing the importance of dengue epidemic management within the district to maintain a safe environment for both local population and tourists.

**Fig. 1:**
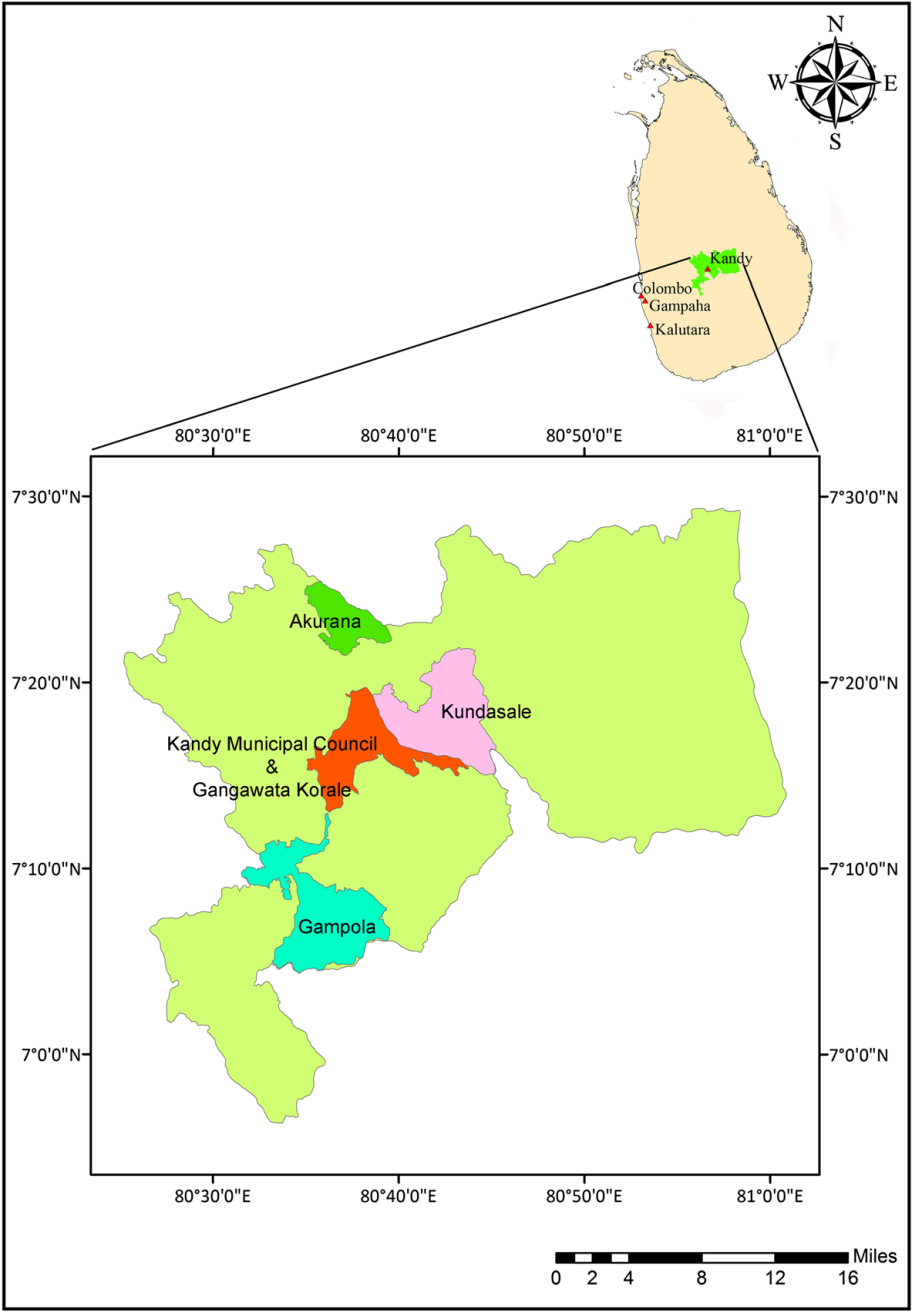
Selected MOH areas in the District of Kandy

Within the period of January to December 2017, Kandy District was the third high risk area for dengue transmission in the country contributing to 7.74% (14,408 cases) of the total dengue cases reported [3]. Of the 23 Medical Officer of Health (MOH) areas in Kandy District, 4 MOH areas that reported the highest number of dengue cases during the period 2010–2015, were selected for the study (Fig. 1). These are Kandy Municipal Council (KMC), Gampola, Akurana and Gangawata Korale (GK).

### Data collection

Entomological surveillance activities were conducted on a monthly basis within the selected four MOH areas during the period January 2016 to August 2017, using standard dipping, siphoning and pipetting methods in accordance with the guidelines recommended by the National Dengue Control Unit, Sri Lanka [26]. Furthermore, random collection of 10 *Aedes* larvae from each positive container (all larvae if the container has < 10 larvae), was followed in entomological surveillance to prevent missing of vector species [27]. Written consent was obtained from the household heads permitting to conduct the entomological surveillance within their homesteads during the specified study period.

Standard morphological keys developed for *Aedes* mosquitoes were used for the identification of *Aedes* larvae during the surveillance [28]. The Premises Index (PI; percentage of houses positive for *Aedes* larvae) and the Breteau Index (BI; number of positive containers with *Aedes* larvae per 100 houses) and Container Index (CI; number of positive containers with *Aedes* larvae per 100 containers) were calculated for each MOH area, based on the WHO guidelines [29]. In addition, the past monthly larval indices corresponding to the period 2010–2015 in the study MOH areas, were obtained from the regional office, Kandy. Furthermore, the number of reported dengue cases from the study MOH areas were collected at monthly level from January 2010 to August 2017.

### Data interpretation and statistical analysis

Pearson’s correlation analysis in SPSS (version 23) was used to evaluate the relationship between different larval indices and the reported dengue cases at different monthly lag periods. Monthly numerical values of each vector index were arranged separately and an order number “m” was assigned for each value (thus for the first entry “m = 1”, for the second entry “m = 2” and so on, till the last event for which m = N). The Gumbel frequency analysis of the series of monthly larval indices intends to obtain a relationship between the magnitude of each larval index value and its probability of exceedance [30, 31]. The probability of exceedance of the event obtained by the use of Gumbel empirical formula is known as the plotting position. Initially, the variance of the data set (*v*) was calculated by using Equation 1, where x_i_ is larval index value, when m = i, $\overline{x}$ is mean of the considering larval index and n is total number of observations [30].

1 $$ Variance(v)={\left(\frac{\varSigma {\left({\mathrm{x}}_{\mathrm{i}}-\overline{\mathrm{x}}\right)}^2}{\mathrm{n}-1}\right)}^{0.5} $$

Subsequently, the reduced variance (*y*) was calculated as shown in Equation 2.

2 $$ Reduced\ variance\ (y)=\frac{\left[\left({x}_i-\overline{x}\right)+\left(0.45\ast v\right)\right]}{0.7797\ast v} $$

The plotting position (P) or the probability of exceedance of each numerical value (different indicators separately) is an exponential function of y as indicated in Equation 3, in accordance with Gumbel frequency distribution [31].

3 $$ \mathrm{P}={\left[{\left(1-e\right)}^{-e}\right]}^{-y} $$

In general, the probability of exceedance of a certain numerical value denotes the number of times or the regularity of which the particular numerical value occurs in the nature. For instance, in Akurana, BI_agp_ of 0.53 can be found 94.27 times among 100 events (months). Graphs indicating the probability of exceedance of the numerical value range of larval indices were developed by plotting probability of exceedance against the magnitude of larval indices. The optimal threshold points for dengue epidemic management were established based on natural exceedance probability of each larval index, followed by a sample back calculation using constructed graphs and the above equations.

## Results

### Seasonality and distribution of vector indices

The temporal variations in the average annual vector indices, with respect to BI values of both *Ae. aegypti* (BI_agp_) and *Ae. albopictus* (BI_alb_), PI and CI during the period January 2010 to August 2017, are illustrated in Additional file 1: Figures S1-S4. It is important to note that there is a seasonal fluctuation pattern of larval density indices in Kandy District. Two major epidemic cycles, *viz* April to July and October to December/January, could be identified annually representing the epidemic peak in June/July and December/January, respectively in each cycle. However, the highest number of cases was reported from the epidemic cycle: April to July, indicating the severity of disease transmission during this period.

### Breteau Index

**Fig. 2:**
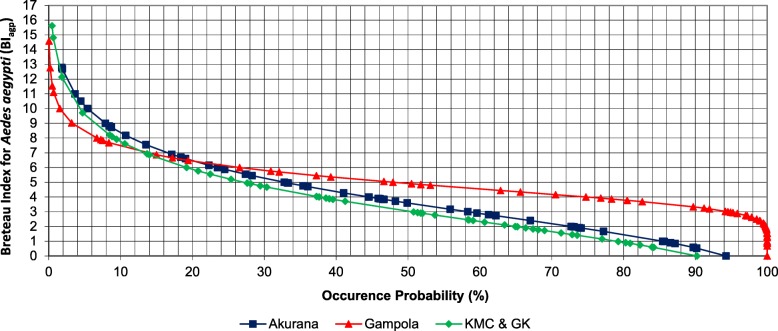
Probability plot for Gumbel frequency analysis of the Breteau Index for *Aedes aegypti* (BI_agp_) in study MOH areas

**Fig. 3:**
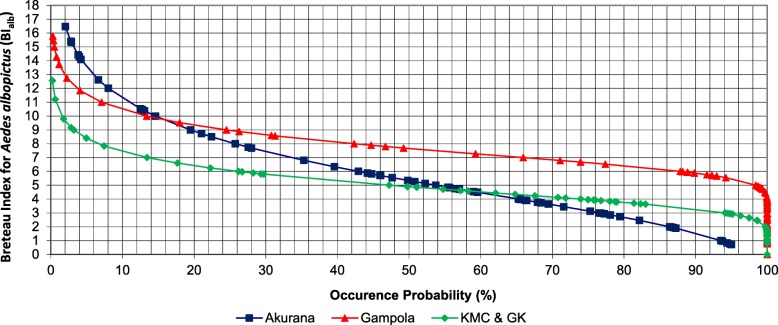
Probability plot for Gumbel frequency analysis of the Breteau Index for *Aedes albopictus* (BI_alb_) in study MOH areas

The highest value of maximum BI_agp_ value (15.62) was recorded in the KMC and GK MOH areas (Fig. 2), while Akurana had the lowest value of maximum BI_agp_ value as 12.14. Akurana MOH area recorded the highest maximum parameter for BI_alb_, (16.46), while KMC and GK denoted the lowest value of maximum BI_alb_ (12.56), as illustrated in Fig. 3. Interestingly, a 0.53 value of BI_agp_ was characterized with a 94.27% exceedance frequency in Akurana. On the contrary, Gampola indicated 1.52 BI_agp_ with a 100% probability of exceedance (Fig. 2).

Meanwhile, a probability of exceedance of 90.21% was shown by a BI_agp_ value of 0.58 in KMC and GK, as the lowest possible positive value for the index within the study period. The BI_alb_ value of 0.72 was identified as the lowest value with a probability of exceedance of 97.67% in Akurana, while 3.76 in Gampola was the lowest with a 100% frequency of exceedance. On the other hand, 0.84 in KMC and GK also showed a probability of exceedance of 100% for BI_alb_ (Fig. 3). In general, Akurana MOH area had relatively high vector densities for both *Ae. aegypti* and *Ae. albopictus* species based on BI, PI and CI indices. Meanwhile, the larval indices were relatively lower in the KMC and GK followed by Gampola MOH area (Fig. 2).

### Threshold values

Four threshold categories were defined for the management of dengue vectors based on the natural occurrence of BI_agp_ and BI_alb_ (Table 1) along with ecofriendly community-based recommendations for different risk stages to ensure effective management of dengue vectors with minimum financial allocations and environmental impacts. The natural probability of exceedance of 85% was selected as the initial threshold value for “low risk” to alert the community. During the “risk” phase (60–85% of exceedance probability), the local community is encouraged to conduct routine cleaning programmes within the locality. In the “moderate risk” phase (40–60 %), intensive vector surveillance, community mobilization strategies and government involved cleaning programmes should be conducted along with focused chemical fogging. Intensive fogging should only be conducted within the final phase of “high risk” (20–0%).

**Table 1:** Recommended Breteau Index for *Aedes aegypti* (BI_agp_) and *Aedes albopictus* (BI_alb_) values based on frequency analysis

Probability of occurrence (%)	BI_agp_ (%)				BI_alb_ (%)				Risk category	Recommended actions
	Akurana	Gampola	KMC and GK	Average	Akurana	Gampola	KMC and GK	Average		
20	6.50	6.40	5.80	6.23	8.80	9.20	6.40	8.13	High risk	Extensive fogging
40	4.30	5.30	3.80	4.47	6.30	8.20	5.30	6.60	Moderate risk	Target oriented fogging
60	2.90	4.50	2.30	3.23	4.45	7.20	4.50	5.38	Risk	Intensive vector surveillance and government involved source reduction programmes
85	1.20	3.6	0.50	1.77	2.15	6.20	3.50	3.95	Low risk	Be alert and motivate source reduction of vector breeding sites

### Premises Index and Container Index

**Fig. 4:**
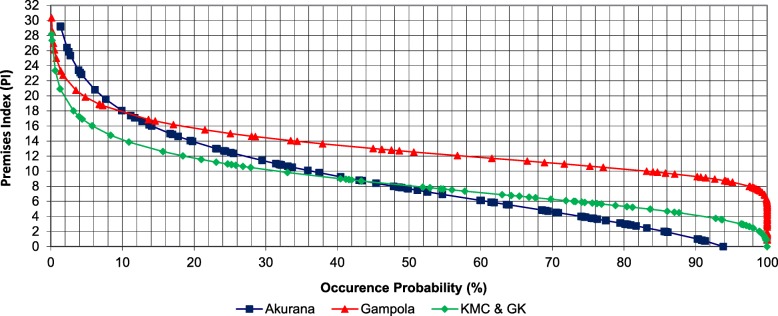
Potential impact of the proposed threshold values for BIA on the number of dengue cases reported within the KMC and GK study MOH area in the period 2011–2014

The highest (30.36) and lowest (0.72) PI values were identified from Gampola and Akurana MOH areas with a probability of exceedance of 0.11% and 93.86%, respectively. The PI in both KMC and GK MOH areas indicated a value of 0.84 with a 100 % probability of exceedance (Fig. 4).

**Fig. 5:**
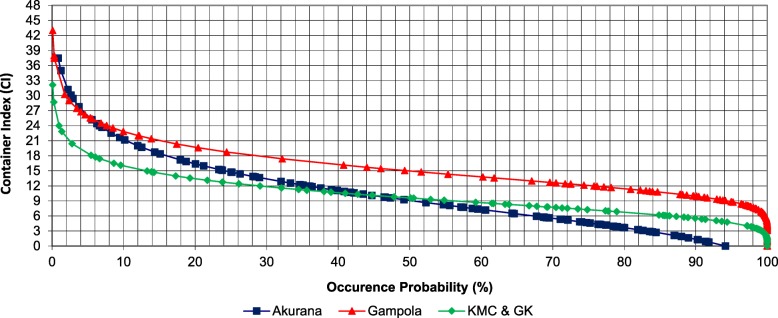
Probability plot for Gumbel frequency analysis of Premises Index (PI) in study MOH areas

Gampola had the highest maximum CI value (43.10) among the study MOH areas. As indicated in Fig. 5, the greatest minimum CI value of 3.86 (characterized by a probability of exceedance of 72.10%) was noted in Gampola, followed by Akurana (0.81) and KMC and GK (0.74). Based on the natural occurrence of both CI and PI, three risk categories were defined as “low risk” (50–75%), “moderate risk” (50–25%) and “high risk” (< 25 %) as indicated in Table 2.

**Table 2:** Recommended Premise Index (PI) and Container Index (PI) values for Kandy based on frequency analysis

Probability of occurrence (%)	PI (%)				CI (%)				Risk category	Recommended action
	Akurana	Gampola	KMC and GK	Average	Akurana	Gampola	KMC and GK	Average		
25	12.45	15.00	11.00	12.82	14.75	18.75	12.40	15.30	High risk	Application of chemical larvicides for potential breeding sites
50	7.70	12.60	8.00	9.43	9.00	15.00	9.58	11.19	Moderate risk	Intensive vector surveillance and government involved source reduction of vector breeding sites
75	3.80	10.70	5.76	6.75	4.60	12.14	7.26	8.00	Low risk	Be alert and motivate source reduction of vector breeding sites

### Association among larval indices and occurrence of dengue epidemics

In all the study MOH areas, *Ae. aegypti* (BI_agy_) dominated the incidence of dengue epidemics, at both 1 and 2 months lag periods with significant (*P* < 0.05) strong Pearson’s correlation coefficients (PC > 0.6). It was interesting to note that the impact of *Ae. albopictus* (BI_alb_) mostly remained negatively correlated and poor (PC < 0.33). In the case of KMC and GK MOH areas, the effect of PI was significant (*P* < 0.05), which denoted moderate negative associations (0.33 > PC < 0.66). The CI always denoted a positive relationship with the number of reported dengue cases in all the study MOH areas. However, the relationships of CI with reported cases were significant only in Akurana (PC > 0.33) at both 1 and 2 month lag periods (Table 3). Therefore, the entomological management of dengue vectors, especially *Ae. aegypti* (BI_agy_), based on the above specified thresholds (Tables 1 and 2) would lead to the management of dengue epidemics within the study MOH areas.

**Table 3:** Results of the Pearson’s correlation analysis for the association among the dengue cases and larval indices at different lag periods in the study areas

Lag period (months)	MOH areas											
	KMC and GK				Akurana				Gampola			
	BI_agp_	BI_alb_	PI	CI	BI_agp_	BI_alb_	PI	CI	BI_agp_	BI_alb_	PI	CI
0	0.359	-0.298	-0.286^a^	0.109	0.297	-0.368	-0.402	0.174	0.335	-0.297	-0.247	0.187
1	0.799^a^	-0.382	-0.524^a^	0.287	0.763^a^	-0.253	-0.357	0.454^a^	0.684^a^	-0.217	-0.428^a^	0.351^a^
2	0.782^a^	-0.346	-0.541^a^	0.357	0.692^a^	-0.124	-0.248	0.387^a^	0.601^a^	-0.089	-0.497^a^	0.204

^a^Indicates significant relationships among the dengue cases and larval indices at different lag periods

**Fig. 6:**
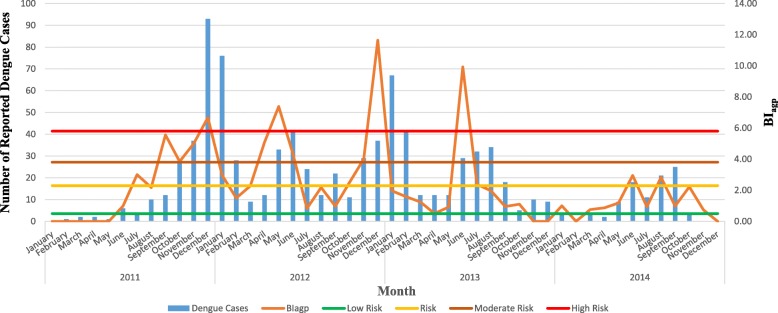
Probability plot for Gumbel frequency analysis of Container Index (CI) in study MOH areas

Figure 6 depicts the temporal variation of reported dengue cases during the period 2011–2014 in KMC and GK MOH areas. For instance, based on observation, controlling the density of *Ae. aegypti* below 2.3 (under risk level) or 3.8 (under moderate risk level), would clearly minimize the severity of the dengue epidemics that occur with a lag period of 1 to 2 months, suggesting that the current thresholds could be utilized in entomological based dengue management (Fig. 6). Furthermore, due to the significant association among the larval indices and the dengue epidemic incidence, the defined thresholds would also be beneficial as alarming tools for dengue outbreaks, whereby health authorities can remain vigilant in implementing vector control efforts before the disease occurrence reaches a high epidemic.

## Discussion

Routine entomological surveillance is essential in identifying high risk areas for dengue, identifying windows for potential dengue epidemics, and to target and implement effective dengue outbreak management efforts. The impact of the geographical scale on the entomological indices has received less attention during dengue transmission studies [4]. Some approaches have made to determine the relationship between *Aedes* population and dengue transmission by different entomological indices that focus on various stages of the mosquito life-cycle: larval [4, 16, 21, 22], pupal [11, 32, 33] and adults [34]. However, these studies have been mostly limited to academic research and have not lead to practical implementation by the vector control authorities in many countries in the world, including Sri Lanka.

Several studies have shown that the larval indices may closely correlate with dengue incidence at a disease outbreak. This suggests that the larval indices provide fairly reliable alerts on forecasting and managing the severity of probable dengue epidemics [4, 18]. In a meta study, Bowman et al. [8] argued on the reliability of quantitative associations between vector indices and dengue transmission. However, some researchers have suggested that the properly defined threshold values for entomological indices reflecting micro level dynamics of disease vector abundance in respective endemic areas, may be proficient in yielding reliable and transparent predictions on upcoming dengue epidemics. However, such attempts are very limited in many countries [4].

Communities defined by administrative boundaries are often considered for the calculation of entomological indices in most countries, without respecting the entomological homogeneity. This may directly influence the overall effectiveness of the entomological indices in reflecting the potential outbreaks of dengue epidemics. Furthermore, several other factors such as herd immunity [19, 20], population characteristics of humans and vectors [17, 35], virus strain [19, 20, 22], and environmental conditions [23, 36] may also affect the relationship between *Aedes* density (reflected by larval indices) and the potential hazard of dengue epidemics [4]. On the other hand, inadequate knowledge, lack of trained staff at regional level, and commitment and satisfaction of field staff involved in field based activities, may also influence the accuracy and quality of entomological surveys. Therefore, all these factors that resemble the local variability should be considered along with the larval parameters in defining entomologically driven threshold values for controlling of dengue epidemics *via* entomological management. Long-term data on seroprevalence of dengue, confirmed dengue cases and herd immunity status of the local populations is limited in the Sri Lankan context. Therefore the current study, focused on derivation of thresholds based on entomological indices for the entomological management of dengue vectors, since it is the only available data source with relatively high precision.

### Breteau Index

In Sri Lanka, the vector control activities of the VCEs are mainly driven based on the findings of BI and PI. However, more attention is drawn to the BI of *Ae. aegypti* (BI_agp_) and *Ae. albopictus* (BI_alb_). Even though, statistically derived thresholds are not practiced, a recent document issued by three national vector controlling institutes in Sri Lanka, has recommended the BI value of 5 as the lowest threshold within which chemical controlling is not required. In addition, a scenario where the BI value ranges from 5 to 20, without cases, has been recommended to be dealt only with breeding place reduction programmes without chemical approaches such as fogging, while scenarios with reported cases or BI > 20, have been recommended for fogging [24].

However, when the dynamics of the above entomological indices within the study areas are considered, it was noted that both BI_aeg_ and BI_alb_ rarely exceed the numerical value of 18. Even though combination of the BI values of both *Ae. aegypti* and *Ae. albopictus* could exceed 20, it may not reflect the actual epidemic potential, since the dominant vector of the dengue epidemic may not solely contribute to the BI. Still, severe epidemics have been reported from the study MOH areas [3]. Therefore, the threshold values that are being used by the VCEs of Sri Lanka, remain relatively less effective and non-applicable. On the other hand, the critical thresholds suggested under the current study at individual larval index level (rather than for combined indices) caters well for the requirement (as indicated in Fig. 6), by stipulating effective cutoffs for vector control.

Several recent studies carried out in Havana, Thailand and Trinidad [4, 36, 37] emphasized that BI and PI could be used as effective indicators for incidence of dengue epidemics. For many years, HI > 1 and BI> 5 have been cast-off as the threshold for dengue high risk in many countries [38]. In another study, BI > 4 has been suggested as the high risk potential, while BI < 1 has been recognized as the low risk threshold [4]. The findings of the current study also suggest similar values for vector controlling in Kandy, where, BI_agp_ > 3.23 and BI_agp_ > 4.47 were suggested as average thresholds for “Risk” and “Moderate risk” categories, respectively.

In reality, it is well known that the *Ae. aegypti* is the primary vector driving the epidemics of dengue [1, 2], while the density of *Ae. albopictus* remains high in most of the areas in Sri Lanka. Hence, relying upon the BI of *Ae. aegypti* (which is the primary vector) could be more effective and capable in managing the dengue epidemics in Sri Lanka, rather than focusing on both indices, due to limitations in financial, technical and human resources. However, appropriate thresholds for BI_alb_ have also been suggested to be used if needed to improve the efficacy of controlling vectors.

In addition, population dynamics of the dengue vectors often change from locality to locality at the regional level due to variations in environmental, meteorological and socio-economic conditions [4, 23, 34, 36]. Therefore, defining the thresholds for vector management irrespective of individual population dynamics and regional variations would be less appropriate and less effective. For instance, situations where BI > 20 is scarce in the majority of the MOH areas, especially in Kandy, although it is a high risk area for dengue at present.

Integrated vector management (IVM) has gained a wider acceptance as the most effective and sustainable approach for controlling of vector borne diseases throughout the world. However, previously introduced threshold guideline in Sri Lanka suggests chemical fogging as the only documented controlling effort for dengue management (if BI > 20 or > 5 with a notable number of cases). Therefore, previously set threshold values to implement other components in the IVM such as source reduction and community involvement are inadequately defined.

In order to remedy these limitations, the present study has suggested different thresholds along with appropriate vector controlling efforts such as motivation for source reduction (organizing of cleaning programmes, awareness programmes) at the low risk level followed by government involved source reduction programmes, intensive vector surveillance activities during the risk phase. In case of moderate risk, target oriented chemical fogging is recommended, rather than waiting for extensive fogging as the only solution (recommended at the high risk level). Hence, the current study addressing the natural population dynamics of the *Aedes* vectors, attempt to link vector surveillance along with community involved IVM, which would be useful in establishing an ecofriendly and community responsive framework for vector control.

### Premises Index and Container Index

Both HI and CI were developed in 1923 aiming to drive the entomological surveillance and also as a measure of the efficacy of implemented vector controlling strategies on the vector populations [39]. Subsequently, the motivation of worldwide vector surveillance of *Aedes* and other related vectors by the World Health Organization (WHO) in late 1960s encouraged many countries to base their routine entomological surveillance on these *Stegomyia* indices [11].

However, critical comments have been raised on the efficacy of both indices, especially regarding CI due to numerous limitations (such as the inability to account for a number of positive containers per house, per area and per person, etc.), regardless of their capability in indicating the types of the most frequent breeding sites in the locality acting as a qualitative tool in directing vector breeding prevention priorities [11]. On the other hand, the PI plays a better role. However, it has yet failed to establish a relationship with number of positive containers per house [11, 34]. If defined appropriately, a set of effective thresholds that address the natural regional variability of the vectors in relation to incidence of dengue epidemics, may compensate for the above shortcomings. Even though VCEs in Sri Lanka consider PI and CI during vector surveillance activities, still no thresholds specific to PI and CI are being practiced by the VCE in vector management approaches.

However, several other countries have defined successful thresholds based upon PI in addition to BI. The Pan American Health Organization has defined three risk levels as low risk (PI < 0.1%), moderate risk (PI 0.1–5%), and high risk (PI > 5%) based on PI [40]. In Salvador, Brazil, sentinel surveillance in 30 areas detected a significant 1.4 times higher sero-incidence when the HI was > 3% [35]. The CI is less used for threshold definition in many countries due to its reduced effectiveness, in reflecting the actual conditions of vector populations with respect to the anthropogenic settings [11]. Nevertheless, the current study also defined three risk threshold levels based on the exceedance probability of the numerical values of PI and CI that were subsequently linked to the IVM approaches. PI > 6.75% was defined as low risk, while PI > 9.43 and PI > 12.82 were defined as moderate and high risk, respectively, as an average.

In a study to determine the vector abundance in six Asian countries including Sri Lanka, Wai et al. [41] found that pupal productivity surveys and identifying the most productive container types, was a better means of targeting interventions, particularly in the wet season. Even though capturing gravid female *Ae. aegypti* female mosquitoes using sticky traps [42] is a very reliable predictor of infestation indices, many countries still use breeding sites such as containers for surveillance. The productive container types can vary between countries, within the country and ecosystems. Incorporating these variables to determine threshold values should result in reliable predictors of epidemic outbreaks, which can be forestalled with targeted intervention by VCE.

### Recommendations and the way forward

Application of a frequency analysis *via* empirical modelling to analyze the natural occurrence frequency of different larval indices and defining critical thresholds based on the frequency of exceedance at the regional level, may be capable of compensating for most factors that deals with the local variability of the epidemics. Hence, similar approaches could be encouraged to be applied in other countries, also in outlining thresholds for entomological indices towards epidemic management at the regional level. Furthermore, it is recommended to use the threshold values defined for specific study areas at the MOH levels, rather than applying the average threshold values defined at a national level.

The limitations in the availability of detailed surveillance data, before, during, and after dengue epidemics can restrict the definition of regional specific threshold values for all the MOH areas in Kandy District. Therefore, the current study suggests average threshold values as a remedy by considering the most high risk MOH areas as a precaution. In depth analysis on the practical applicability of the above defined thresholds by the relevant VCEs, is highly recommended subjecting to calibration and re-arrangement of the defined thresholds in order to achieve better accuracy in predictions.

## Conclusions

Among the study MOH areas, Akurana had a relatively higher BI density of *Ae. aegypti* and *Ae. albopictus* (in terms of BI_agp_ and BI_alb_), while PI and CI also remained relatively higher in Akurana MOH. In contrast, Gangawata Korale MOH area had the lowest larval indices. Based on the population dynamics of *Ae. aegypti*, four risk thresholds could be defined for Kandy as low risk (BI_agp_ > 1.77), risk (BI_agp_ > 3.23), moderate risk (BI_agp_ > 4.47) and high risk. In addition, PI > 6.75% was defined as low risk, while PI > 9.43 and PI > 12.82 were defined as moderate and high risk, respectively, as an average. Application of the threshold values recommended for *Ae. aegypti* (primary vector for dengue) along with cut-off values for PI (accounting for both *Ae. aegypti* and *Ae. albopictus)*, could be recommended to control epidemic outbreaks, while addressing the limitations in financial, technical and human resources of Sri Lanka for vector controlling activities.

## Additional file

Additional file 1: **Figure S1.** Temporal variations in the monthly average Breteau Index for *Aedes aegypti* (BI_agp_) each MOH area in the District of Kandy (2010 to 2017). **Figure S2.** Temporal variations in the monthly average Breteau Index for *Aedes albopictus* (BI_alb_) in each MOH area in the District of Kandy (2010 to 2017). **Figure S3.** Temporal variations in the monthly average Container Index (CI) in each MOH area in the District of Kandy (2010 to 2017). **Figure S4.** Temporal variations in the monthly average Premise Index (PI) in each MOH area in the District of Kandy (2010 to 2017). (DOCX 34 kb)

## Abbreviations

BI: Breteau Index
BI_agp_: Breteau Index for *Ae. aegypti*
BI_alb_: Breteau Index for *Ae. albopictus*
CI: Container Index
GK: Gangawata Korale
IVM: integrated vector management
KMC: Kandy Municipal Council
PI: Premises Index
MOH: Medical Officer of Health
VCE: vector controlling entities

## References

[1] Bhatt S, Gething PW, Brady OJ, Messina JP, Farlow AW, Moyes CL (2013). The global distribution and burden of dengue. Nature..

[2] Lambrechts L, Scott TW, Gubler DJ (2010). Consequences of the expanding global distribution of *Aedes albopictus* for dengue virus transmission. PLoS Negl Trop Dis..

[3] Epidemiology Unit, Ministry of Health. Dengue update. 2018 http://www.epid.gov.lk/web/index.php?Itemid=448%23. Accessed 25 July 2018.

[4] Sanchez L, Vanlerberghe V, Alfonso L, del Carmen Marquetti M, Guzman MG, Bisset J (2006). *Aedes aegypti* larval indices and risk for dengue epidemics. Emerg Infect Dis..

[5] Guzmán MG, Kouri G (2002). Dengue: an update. Lancet Infect Dis..

[6] World Health Organization (2009). Dengue: guidelines for diagnosis, treatment, prevention and control.

[7] Reiter P, Gubler DJ, Ooi EE, Vasudevan S, Farrar J (2014). Surveillance and control of urban dengue vectors. Dengue and dengue hemorrhagic fever.

[8] Bowman LR, Runge-Ranzinger S, McCall PJ (2014). Assessing the relationship between vector indices and dengue transmission: a systematic review of the evidence. PLoS Negl Trop Dis..

[9] Getis A, Morrison AC, Gray K, Scott TW. Characteristics of the spatial pattern of the dengue vector, *Aedes aegypti*, in Iquitos, Peru. In: Anselin L, Rey S, editors. Perspectives on spatial data analysis. Berlin-Heidelberg: Springer; 2010. p. 251–306.

[10] Maciel-de-Freitas R, Valle D (2014). Challenges encountered using standard vector control measures for dengue in Boa Vista, Brazil. Bull World Health Organ..

[11] Focks DA (2003). A review of entomological sampling methods and indicators for dengue vectors.

[12] Cromwell EA, Stoddard ST, Barker CM, Van Rie A, Messer WB, Meshnick SR (2017). The relationship between entomological indicators of *Aedes aegypti* abundance and dengue virus infection. PLoS Negl Trop Dis..

[13] Chen SC, Liao CM, Chio CP, Chou HH, You SH, Cheng YH (2010). Lagged temperature effect with mosquito transmission potential explains dengue variability in southern Taiwan: insights from a statistical analysis. Sci Total Environ..

[14] Tran A, Deparis X, Dussart P, Morvan J, Rabarison P, Remy F (2004). Dengue spatial and temporal patterns, French Guiana, 2001. Emerg Infect Dis..

[15] Pérez Martínez TT, Íñiguez Rojas L, Sánchez Valdés L, Remond NR (2003). Vulnerabilidad espacial al dengue: Una aplicación de los sistemas de información geográfica en el municipio Playa de Ciudad de La Habana. Rev Cub Salud Pública..

[16] Moore CG, Cline BL, Ruiz-Tibén E, Lee D, Romney-Joseph H, Rivera-Correa E (1978). *Aedes aegypti* in Puerto Rico: environmental determinants of larval abundance and relation to dengue virus transmission. Am J Trop Med Hyg..

[17] Espinoza Gómez F, Hernández Suárez CM, Coll Cárdenas R (2001). Factors that modify the larval indices of *Aedes aegypti* in Colima, Mexico. Rev Panam Salud Pública..

[18] Pontes RJ, Freeman JO, Oliveira-Lima JW, Hodgson JC, Spielman AN (2000). Vector densities that potentiate dengue outbreaks in a Brazilian city. Am J Trop Med Hyg..

[19] Yew YW, Ye T, Ang LW, Ng LC, Yap G, James L (2009). Seroepidemiology of dengue virus infection among adults in Singapore. Ann Acad Med Singapore..

[20] Goh KT, Ng SK, Chan YC, Lim SJ, Chua EC (1987). Epidemiological aspects of an outbreak of dengue fever/dengue haemorrhagic fever in Singapore. Southeast Asian J Trop Med Public Health..

[21] Chan YC, Chan KL, Ho BC (1971). *Aedes aegypti* (L.) and *Aedes albopictus* (Skuse) in Singapore City: 1. Distribution and density. Bull World Health Organ..

[22] Neff JM, Morris L, Gonzalez-alcover R, Coleman PH, Lyss SB, Negron H (1967). Dengue fever in a Puerto Rican community. Am J Epidemiol..

[23] Scott TW, Morrison AC, Takken W, Scott TW (2004). *Aedes aegypti* density and the risk of dengue-virus transmission. Ecological aspects for application of genetically modified mosquitoes.

[24] Anti-Malaria Campaign Directorate (2016). Guidelines for interpretation of data from vector surveillance and chemical vector control activities.

[25] Kandy District Secretariat, Sri Lanka. About us/ Kandy District Secretariat. 2012. http://www.kandy.dist.gov.lk. Accessed 25 March 2018.

[26] National Dengue Control Unit, Sri Lanka. Guidelines for *Aedes* vector surveillance and control. Colombo: National Dengue Control Unit, Sri Lanka; 2016. p. 39–44.

[27] Kusumawathie PHD, Jayasooriya GAJSK, Wickremasinghe AR. Sensitivity of different larval collection methods in dengue vector surveillance in the Kandy and Nuwara Eliya district. In: Proceedings of the 62nd Annual Sessions, part 1, abstracts of 2006. Colombo: Sri Lanka Association for the Advancement of Science; 2006. p. 1–2.

[28] Rueda LM (2004). Pictorial keys for the identification of mosquitoes (Diptera: Culicidae) associated with dengue virus transmission.

[29] World Health Organization (1995). Western Pacific education in action series No.8.

[30] Gumbel EJ (1941). The return period of flood flows. Ann Math Statist..

[31] Das MM, Saikia MD (2009). Hydrology.

[32] Focks DA, Chadee DD (1997). Pupal survey: an epidemiologically significant surveillance method for *Aedes aegypti*: an example using data from Trinidad. Am J Trop Med Hyg..

[33] Strickman D, Kittayapong P (2003). Dengue and its vectors in Thailand: calculated transmission risk from total pupal counts of *Aedes aegypti* and association of wing-length measurements with aspects of the larval habitat. Am J Trop Med Hyg..

[34] Rodriguez-Figueroa L, Rigau-Perez JG, Suarez EL, Reiter P (1995). Risk factors for dengue infection during an outbreak in Yanes, Puerto Rico in 1991. Am J Trop Med Hyg..

[35] Teixeira MD, Barreto ML, Costa MD, Ferreira LD, Vasconcelos PF, Cairncross S (2002). Dynamics of dengue virus circulation: a silent epidemic in a complex urban area. Trop Med Int Health..

[36] Thammapalo S, Chongsuvivatwong V, Geater A, Dueravee M (2008). Environmental factors and incidence of dengue fever and dengue haemorrhagic fever in an urban area, Southern Thailand. Epidemiol Infect..

[37] Chadee DD, Williams FLR, Kitron UD (2005). Impact of vector control on a dengue fever outbreak in Trinidad, West Indies, in 1998. Trop Med Int Health.

[38] Tun-Lin W, Kay BH, Barnes A, Forsyth S (1996). Critical examination of *Aedes aegypti* indices: correlations with abundance. Am J Trop Med Hyg..

[39] Connor ME, Monroe WM (1923). *Stegomyia* indices and their value in yellow fever control. Am J Trop Med Hyg..

[40] Pan American Health Organization (PAHO) (1994). Dengue and dengue hemorrhagic fever in the Americas. Guidelines for prevention and control. Scientific publication no. 548.

[41] Wai KT, Arunachalam N, Tana S, Espino F, Kittayapong P, Abeyewickreme W (2012). Estimating dengue vector abundance in the wet and dry season: implications for targeted vector control in urban and peri-urban Asia. Pathog Glob Health..

[42] da Cruz Ferreira DA, Degener CM, de Almeida Marques-Toledo C, Bendati MM, Fetzer LO, Teixeira CP, Eiras ÁE. Meteorological variables and mosquito monitoring are good predictors for infestation trends of *Aedes aegypti*, the vector of dengue, chikungunya and Zika. Parasit Vectors. 2017;10:78. 10.1186/s13071-017-2025-8 PMC5307865 28193291

