# Supplementary material for: Empirical optimization of risk thresholds for dengue: an approach towards entomological management of Aedes mosquitoes based on larval indices in the Kandy District of Sri Lanka
Source: Parasit Vectors. 2018 Jun 28;11:368. doi: 10.1186/s13071-018-2961-y (PMC6022305; doi:10.1186/s13071-018-2961-y)
Supplement: Supplementary file 1 — Figure S1. Temporal variations in the monthly average Breteau Index for Aedes aegypti (BIagp) each MOH area in the District of Kandy (2010 to 2017). Figure S2. Temporal variations in the monthly average Breteau Index for Aedes albopictus (BIalb) in each MOH area in the District of Kandy (2010 to 2017). Figure S3. Temporal variations in the monthly average Container Index (CI) in each MOH area in the District of Kandy (2010 to 2017). Figure S4. Temporal variations in the monthly average Premise Index (PI) in each MOH area in the District of Kandy (2010 to 2017). (DOCX 34 kb) [file 13071_2018_2961_MOESM1_ESM.docx]

**Additional file 1**

**Figure S1**. Temporal variations in the monthly average Breteau Index for *Aedes aegypti* (BI_agp_) each MOH area in the district of Kandy (2010 to 2017).

**Figure S2**. Temporal variations in the monthly average Breteau Index for *Aedes albopictus* (BI_alb_) in each MOH area in the district of Kandy (2010 to 2017).

**Figure S3**. Temporal variations in the monthly average Container Index (CI) in each MOH area in the district of Kandy (2010 to 2017).

**Figure S4**. Temporal variations in the monthly average Premise Index (PI) in each MOH area in the district of Kandy (2010 to 2017).
